# Synthesis and Docking Analysis of New Heterocyclic System N^1^, N^4^-bis (2-chloroquinolin-3-yl) methylene) benzene-1, 4-diamine as Potential Human AKT1 Inhibitor

**Published:** 2016

**Authors:** Sohrab Ghanei, Jalil Lari, Hossein Eshghi, Mohammad Saadatmandzadeh

**Affiliations:** a *Department of Chemistry, School of sciences, Ferdowsi University of Mashhad, Mashhad, Iran.*; b *Department of Chemistry, School of sciences, Payam Nour University of Mashhad, Mashhad, Iran.*

**Keywords:** AKT1 Inhibitors, Cancer, Docking Analysis, Heterocyclic compound, Quinoline derivatives

## Abstract

In recent years, the chemistry of 2-chloroquinoline-3-carbaldehydes have received considerable attention owing to their synthetic and effective biological importance which exhibits a wide variety of biological activity, N^1^,N^4^-bis((2-chloroquinolin-3-yl)methylene)benzene-1,4-diamine derivatives that synthesized from 2-chloroquinoline-3-carbaldehydes may have biological effects. As the inhibitor of AKT1 (RAC-alpha serine/threonine-protein kinase is an enzyme that in humans is encoded by the AKT1), the aforementioned compounds may have implication in preventing complications of cancers.

A group of N^1^, N^4^-bis ((2-chloroquinolin-3-yl) methylene) benzene-1, 4-diamine derivatives (3a-3i) (H, 6-Me, 6-OMe, 6-OEt, 6-Cl, 7-Me, 6-Et, 6-Isopropyl, 7-Cl) were synthesized, and theoretically evaluated for their inhibitory as Potential Human AKT1 Inhibitors via docking process. The docking calculation was done in GOLD 5.2.2 software using Genetic algorithm.

Compounds 3b (6-Me) and 3d (6-OEt) showed the best inhibitory potency by GOLD score value of 113.76 and 107.58 respectively.

Some of the best models formed strong hydrogen bonds with Asn 49, Lys 220, Ser 157, Arg 225 and Trp 76 via quinoline moiety and nitrogen of quinolone ring (Figure 1.). pi-pi interaction between Lys 220, Trp 76, Tyr 224, Arg 225, Ile 80, and Asn 49 quinoline moiety was one of the common factor in enzyme-inhibitor junction.

It was found that both hydrogen bonding and hydrophobic interactions are important in function of biological molecules, especially for inhibition in a complex.

## Introduction

AKT1/PKBa (protein kinase B) is a serine/threonine kinase that belongs to the AKT family AKT1 (RAC-alpha serine/threonine-protein kinase is an enzyme that in humans is encoded by the AKT1), also known as «AKT» or protein kinase B (PKB) is an important molecule in mammalian cellular signaling. In humans, there are three genes in the «AKT family»: AKT1, AKT2, and AKT3. These enzymes are members of the serine/threonine-specific protein kinase family (1).

AKT1 is activated in cells in response to diverse stimuli such as hormones, growth factors and extracellular matrix components and is involved in glucose metabolism, transcription, survival, cell proliferation, angiogenesis, and cell motility (1, 2). AKT1 is frequently overexpressed and activate in many types of human cancers including cancers of colon, breast, brain, pancreas and prostate as well as lymphomas and leukemias (3, 4).

Docking is an important in the study of protein ligand interaction properties such as binding energy, geometry complementarity, hydrogen bond donor acceptor, hydrophobicity, electron distribution and polarizability thus it plays a major role in the drug discovery for the identification of suitable molecular scaffold and distinguishing selectivity for the target protein (5).

GOLD, the first algorithm to be evaluated on a large data set of complex posse, an empirical free energy scoring function that estimates the free energy of binding permitting inhibition constant for protein ligand complex. It is a package of program for structure visualization and manipulation for docking, the post processing and visualization of the results (6). The objective of the present work is to study the in silico of AKT1inhibitory activity of some new synthetic N^1^, N^4^-bis ((2-chloroquinolin-3-yl) methylene) benzene-1, 4-diamine.

Quinoline and its derivatives have always attracted both synthetic and biological chemists because of its diverse chemical and pharmacological properties (7). For example quinine has been used for the treatment of malaria (8), dynemicin A and streptonigrin, naturally occurring members of the class of antitumor antibiotic (9, 10). According to our literature review we find that compounds containing quinoline (11-14), have been reported to exhibit antiinflammatory activity (15). To study the combined effect of these two moieties (quinoline and diimine) in a single network, there is an interest in the synthesis of N^1^, N^4^-bis ((2-chloroquinolin-3-yl) methylene) benzene-1, 4-diamine (3a-3i). Therefore, we studied our compound as a potential inhibitor for AKT1 enzyme.

## Experimental

*Materials and Methods*

The products were characterized by spectroscopic data (IR, ^1^H NMR, elemental analyses). The purity determinations of the products were accomplished by TLC on silica gel polygram STL G/UV 254 plates. Melting points were determined with an Electrothermal Type 9100 melting point apparatus. Elemental analyses were made by a Thermo Finning Flash EA1112 CHNO-S analyzer and agreed with the calculated values. The FTIR spectra were recorded on an Avatar 370 FTIR Therma Nicolet spectrometer. The NMR spectra were recorded on a Bruker Avance 100 and 400 MHz instrument in CDCl_3 _and DMSO. 

*General experimental procedure for the preparation of N* ^1^ *, N* ^4^ *-bis ((2-chloroquinolin-3-yl) methylene) benzene-1, 4-diamine (3a-i)* ***.***

A mixture of 2-chloroquinoline-3-carbaldehydes (3 mmol, 0.5734g) (1), benzene-1,4-diamine (1.5 mmol, 0.1622 g) (2), few drops glacial acetic acid and 40 mL EtOH in a 100 mL flask was stirred at reflux for 4 h. After completion of the reaction (monitored by TLC, ethyl acetate/n-hexane, 1/1), the resulting solid was separated by filtration, and recrystallized from ethanol to afford pure product.

*N* ^1^ *, N* ^4^ *-Bis ((2-chloroquinolin-3-yl) methylene) benzene-1, 4-diamine (3a)*

Yield: 72%; mp 195 °C; IR (KBr) υcm^-1^: 3000-3300 (CH aromatic), 2952 (CH aliphatic), 1616 (C=N imine), 1557 (C=N quinoline), 1520 (C=C quinoline), 1500 (C=C phenyl), 1057 (C-Cl quinolone ring). ^1^H NMR (400 MHz, CDCl_3_, 25 °C, ppm) δ: 7.46 (s, 4H, meddle benzene ring), 7.65 (m, 2H, H7), 7.84 (m, 2H, H6), 8.09 (d, 2H, J=8.4, H5), 8.10 (dd, 2H, J=8.8, 0.8, H8), 9.09(s, 2H, HC=N), 9.10(s, 2H, H4). Anal. Calcd. for C_26_H_16_Cl_2_N_4_: C, 68.58; H, 3.54; Cl,15.57; N, 12.30; Found: C, 68.54; H, 3.57; N, 12.28. 

*N* ^1^ *, N* ^4^ *-Bis ((2-chloro-6-methylquinolin-3-yl) methylene) benzene-1, 4-diamine (3b)*

Yield: 70%; mp 212-215 °C; IR (KBr) υ cm^-1^: 3100 (CH aromatic), 2900-3000 (CH aliphatic), 1619 (C=N imine), 1580 (C=N quinoline), 1525 (C=C quinoline), 1504 (C=C phenyl), 1059 (C-Cl quinolone ring). ^1^H NMR (400 MHz, DMSO, 25 °C, ppm) δ: 2.46 (s, 6H, CH_3_), 6.36 (s, 4H, meddle benzene ring), 7.24 (d, 2H, J=8.75, H7), 7.86 (d, 2H, J=8.75, H8), 7.88 (s, 2H, H5), 7.95 (s, 2H, HC=N), 8.87 (s, 2H, H4). Anal. Calcd. for C_28_H_20_Cl_2_N_4_: C, 69.57; H, 4.17; Cl,14.67; N, 11.59; Found: C, 69.59; H, 4.20; N, 11.62.

*N* ^1^ *, N* ^4^ *-Bis ((2-chloro-6-methoxyquinolin-3-yl) methylene) benzene-1, 4-diamine (3c)*

Yield: 69%; mp 218-220 °C; IR (KBr) υ cm^-1^: 3100-3200 (CH aromatic), 2900-3000 (CH aliphatic), 1615 (C=N imine), 1572 (C=N quinoline), 1518 (C=C quinoline), 1498(C=C phenyl), 1234(C-O), 1055(C-Cl quinolone ring). ^1^H NMR (400 MHz, DMSO, 25 °C, ppm) δ: 3.01 (s, 6H, CH_3_), 6.37 (s, 4H, meddle benzene ring), 6.61 (d, 2H, J=8.75, H7), 7.22 (d, 2H, J=8.75, H8), 7.62 (s, 2H, H5), 7.88 (s, 2H, HC=N), 8.87 (s, 2H, H4). Anal. Calcd. for C_28_H_20_Cl_2_N_4_O_2_: C, 65.25; H, 3.91; Cl,13.76; N, 10.87; Found: C, 65.21; H, 3.90; N, 10.89.

*N* ^1^ *, N* ^4^ *-Bis ((2-chloro-6-ethoxyquinolin-3-yl) methylene) benzene-1, 4-diamine (3d)*

Yield: 74%; mp 196 °C; IR (KBr) υ cm^-1^: 3100-3150 (CH aromatic), 2895-2950 (CH aliphatic), 1600 (C=N imine), 1570 (C=N quinoline), 1520 (C=C quinoline), 1500 (C=C phenyl), 1234 (C-O), 1057 (C-Cl quinolone ring). ^1^H NMR (400 MHz, DMSO, 25 °C, ppm) δ: 1.38 (t, 6H, J=6.75, CH_3 _), 2.74 (q, 4H, J=6.75, CH_2_), 6.39 (s, 4H, meddle benzene ring), 6.61 (d, 2H, J=8.5, H7), 7.21 (d, 2H, J=8.5, H8), 7.45 (s, 2H, H5), 7.60 (s, 2H, HC=N), 8.88 (s, 2H, H4). Anal. Calcd. for C_30_H_24_Cl_2_N_4_O_2_: C, 66.30; H, 4.45; Cl,13.05; N, 10.31; Found: C, 66.29; H, 4.41; N, 10.28.

*N1, N4-Bis ((2, 6 -dichloroquinolin-3-yl) methylene) benzene-1, 4-diamine (3e)*

Yield: 74%; mp 196-198 °C; IR (KBr) υ cm^-1^: 2900-3300 (CH aromatic), 2862 (CH aliphatic), 1620 (C=N imine), 1573 (C=N quinoline), 1519 (C=C quinoline), 1500 (C=C phenyl), 1055(C-Cl quinolone ring). ^1^H NMR (400 MHz, DMSO, 25 °C, ppm) δ: 6.42 (s, 4H, meddle benzene ring), 6.62 (d, 2H, J=8.75, H7), 7.86 (s, 2H, HC=N), 7.90 (d, 2H, J=8.75, H8), 8.37 (s, 2H, H5), 8.88 (s, 2H, H4). Anal. Calcd. For C_26_H_14_Cl_4_N_4_: C, 59.57; H, 2.69; Cl, 27.05; N, 10.69; Found: C, 59.60; H, 2.71; N, 10.66.

*N* ^1^ *, N* ^4^ *-Bis ((2-chloro-7-methylquinolin-3-yl) methylene) benzene-1, 4-diamine (3f)*

 Yield: 70%; mp 198 °C; IR (KBr) υ cm^-1^: 3110-3210 (CH aromatic), 2950 (CH aliphatic), 1612 (C=N imine), 1585 (C=N quinoline), 1519 (C=C quinoline), 1489 (C=C phenyl), 1057 (C-Cl quinolone ring). ^1^H NMR (400 MHz, CDCl_3_, 25 °C, ppm) δ: 2.60(s, 6H, CH_3_), 7.42 (s, 4H, meddle benzene ring), 7.59 (s, 2H, H8), 7.44 (d, 2H. J=8.25, H6), 7.84 (d, 2H, J=8.25, H5), 7.88 (s, 2H, HC=N), 9.02 (s, 2H, H4). Anal. Calcd. for C_28_H_20_Cl_2_N_4_: C, 69.57; H, 4.17; Cl, 14.67; N, 11.59; Found: C, 69.52; H, 4.12; N, 11.61.

*N* ^1^ *, N* ^4^ *-Bis ((2-chloro-6-ethylquinolin-3-yl) methylene) benzene-1, 4-diamine (3g)*

Yield: 69%; mp 190 °C; IR (KBr) υ cm^-1^: 3000-3100 (CH aromatic), 2900-3000 (CH aliphatic), 1610 (C=N imine), 1583 (C=N quinoline), 1517 (C=C quinoline), 1489 (C=C phenyl), 1061 (C-Cl quinolone ring). ^1^H NMR (400 MHz, CDCl_3_, 25 °C, ppm) δ: 1.35 (t, 6H, J=7.5, CH_3_), 2.85 (q, 4H, J=7.5, CH_2_), 7.42 (s, 4H, meddle benzene ring), 7.67 (d, 2H, J=8.5, H7), 7.96, (d, 2H, J=8.5, H8), 7.73 (s, 2H. H5), 8.42 (s, 2H, HC=N), 9.02 (s, 2H, H4). Anal. Calcd. for C_30_H_24_Cl_2_N_4_: C, 70.45; H, 4.73; Cl, 13.86; N, 10.59; Found: C, 70.47; H, 4.70; N, 10.94.

*N* ^1^ *, N* ^4^ *-Bis ((2-chloro-6-isopropylquinolin-3-yl) methylene) benzene-1, 4-diamine (3h)*

Yield: 68%; mp 200 °C; IR (KBr) υ cm^-1^: 3100-3200 (CH aromatic), 2800-3000 (CH aliphatic), 1615 (C=N imine), 1581 (C=N quinoline), 1515 (C=C quinoline), 1500 (C=C phenyl), 1064 (C-Cl quinolone ring). ^1^H NMR (400 MHz, CDCl_3_, 25 °C, ppm) δ: 1.36 (d, J=6.75, 12H, CH_3_), 3.10 (m, 2H, CH), 7.43 (s, 4H, meddle benzene ring), 7.73 (d, 2H, J=8.75, H7), 7.74 (s, 2H, H5), 7.42 (s, 2H, HC=N), 7.99, (d, 2H, J=8.75, H8), 9.04 (s, 2H, H4). Anal. Calcd. for C_32_H_28_Cl_2_N_4_: C, 71.24; H, 5.23; Cl, 13.14; N, 10.38; Found: C, 71.24; H, 5.20; N, 10.36.

*N* ^1^ *, N* ^4^ *-Bis ((2, 7-dichloroquinolin-3-yl) methylene) benzene-1, 4-diamine (3i)*

Yield: 75%; mp 195 °C; IR (KBr) υ cm^-1^: 3000-3200 (CH aromatic), 2907-3005 (CH aliphatic), 1610 (C=N imine), 1583 (C=N quinoline), 1519 (C=C quinoline), 1477(C=C phenyl), 1060(C-Cl quinolone ring). ^1^H NMR (400 MHz, DMSO, 25 °C, ppm) δ: 6.48 (s, 4H, meddle benzene ring), 7.70 (d, 2H, J=8.75, H6), 8.24 (d, 2H, J=8.75, H5), 8.04 (s, 2H, H8), 8.85 (s, 2H, HC=N), 8.82 (s, 2H, H4). Anal. Calcd. For C_26_H_14_Cl_4_N_4_: C, 59.57; H, 2.69; Cl, 27.05; N, 10.69; Found: C, 59.55; H, 2.69; N, 10.72.

*Structure optimization*

Three dimensional structures of the compounds 3a-i were simulated in Hyper Chem7.5 using MM+ method (RMS gradient = 0.1 kcal mol^-1^) (HyperChem® Release 7, Hypercube Inc., http://www.hyper.com/). In the second optimization, output files were minimized under Semi empirical AM1 methods (Convergence limit = 0.01; Iteration limit = 50; RMS gradient = 0.1 kcal mol^-1^; Polak-Ribiere optimizer algorithm) (16, 17).

Crystal structures of AKT1 (EC.2.7.11.1) were retrieved from RCSB Protein Data Bank (PD B entry: 3O96).

*Molecular docking*

Docking was carried out using GOLD 5.2 (Genetic optimization for Ligand Docking) software based on the Gold Score fitness function, that uses the Genetic algorithm (GA). All water molecules and hetero atoms were omitted from the protein to evaluate the two scoring functions in GOLD. For each of the 25 independent GA runs, a maximum number of 100000 GA functions were established on a set of five groups with a population size of 100 individuals. Mutation, migration and operator weights for crossover were set to 95, 10, and 95, respectively. Default cutoff values of 4.0 A for van der Waals distance and 2.5 A (dH-X) for hydrogen bonds were employed. When the top three solutions achieved RMSD values enough 1.5 A, GA docking was terminated. The RMSD values for the docking computations are based on the RMSD matrix of the ranked solutions and observed that the best ranked solutions were always among the first 50 GA runs, and further analyzing of the conformation of molecules performed on the best fitness score. The docking procedure was validated by redocking of tolrestat to the AKT1 crystal structure 3O96.

**Figure 1:**
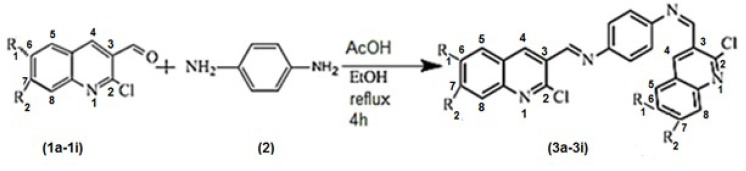
The best docked structure of 3b in the active site pocket of AKT1 (PDB entry: 3O96) in stick (left) and solvent surface (right) views.

**Scheme1:**
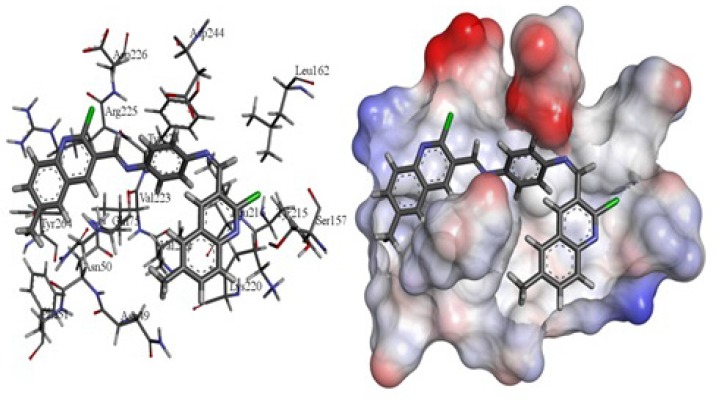
R_1_= H, Me, OMe, OEt, Cl, Et, Isopropyl R_2_= Me, Cl

**Table1:** Synthesis of N^1^, N^4^-bis ((2-chloroquinolin-3-yl) methylene) benzene-1, 4-diamine derivatives

**mp °C**	**Yield (%)** ^a^	**R**	**Product**
195	72	H	**3a**
212-215	70	6-Me	**3b**
218-220	69	6-OMe	**3c**
196	68	6-OEt	**3d**
196-198	74	6-Cl	**3e**
198	70	7-Me	**3f**
190	69	6-Et	**3g**
200	68	6-Isopropyl	**3h**
195	75	7-Cl	**3i**

**Table 2:** gold score, free energy of binding, Estimated inhibitory constant (Ki) and amino acids involved in hydrogen binding with synthetic compounds

**Residues involved in hydrogen binding**	**Ki**	**∆G (kJ/mol)**	**Gold Score**	**compound**
Lys 220, Trp 76	4.16817E-05	-25	96.57	**3a**
Trp 76, Asn 49	1.65321E-06	-33	113.76	**3b**
Tyr 224, Arg 225, Trp 76	1.51422E-05	-27.51	98.19	**3c**
Tyr 224, Trp 76, Ile 80, Arg 225	1.15094E-05	-28.19	107.58	**3d**
Trp 76, Ile 80, Arg 225	9.53038E-05	-22.95	95.81	**3e**
Trp 76, Asn 49	2.86156E-06	-31.64	100.98	**3f**
Lys 220, Asn 49, Trp 76	0.000113815	-22.51	105.16	**3g**
Lys 220, Trp 76, Asn 49	3.56247E-09	-48.22	104.44	**3h**
Trp 76, Tyr 224	3.39728E-08	-42.63	102.05	**3i**
Tyr 76, Tyr 264, Arg 225, Lys 220	1.64349E-8	-44.43	116.02	**Co crystall**

## Results

*Chemistry*

To formation the products (3a-3i), the reaction took place between 2-chloroquinoline-3- carbaldehydes (1a-1i) and benzene-1, 4-diamine (2) as the starting materials in ethanol under reflux condition, Scheme1. The reaction was catalysed with acetic acid.

The completion of the reaction was monitored by TLC, and the disappearance of the starting material was observed within 4 h. 

In order to study the generality of this method, we extended our studies to synthesis of some N^1^, N^4^-bis ((2-chloroquinolin-3-yl) methylene) benzene-1, 4-diamine derivatives (3a-3i). The reactions proceeded very efficiently in relatively high yields as showed in Table 1. All the products were characterized and confirmed by their spectroscopic and elemental analysis data.

*a) Isolated yield*

*Docking*

The receptor 3O96 is a complex structure of enzyme. The possible active site was identified using Accelrys DS Visualizer. Eight active site residues as Trp 80, Ile 84, Cer 205, Lys 268, Tyr 272, Ile 290, Asp 292 andCys 296 were found. Therefore it is chosen as a most biologically favorable site for docking. Some of the best models formed strong hydrogen bonds with Asn 49, Lys 220, Ser 157, Arg 225 and Trp 76 via quinoline moiety and nitrogen of quinolone ring (Figure 1.). pi - pi interaction between Lys 220, Trp 76, Tyr 224, Arg 225, Ile 80, and Asn 49 quinoline moiety was one of the common factor in enzyme-inhibitor junction. Amongst the synthetic compounds, 3e showed the lowest score while 3b possessed the highest score. The estimated inhibitory constant of the docked compounds are outlined in table. 

## Discussion

The complete spectral and elemental analytical data of the products confirmed the formation of new N^1^, N^4^-bis ((2-chloroquinolin-3-yl) methylene) benzene-1, 4-diamine derivatives (3a-3i). The ^1^H NMR spectrum of 3a consist of three singlet signals at δ 7.46, 9.09, 9.10 due to one aromatic and one imine hydrogen and one hydrogen of quinolone ring that posse nitrogen respectively. The doublet signal at δ 8.09 due to the hydrogen on the aromatic ring of quinolines and three multiple for the three hydrogens (on the aromatic ring of quinolines at δ 7.65, 7.84 and 8.10 ppm) were confirmed to the structure. The mass spectrum shows the molecular ion at 454 *m/z*. Also the absence of the stretching vibration band at 1689 cm^-1^ due to the carbonyl group (C=O) in IR spectrum confirmed the structure of 3a. The final proof for this structure received by the results of elemental analysis that was in good agreement calculated data. 

Docking of N^1^, N^4^-bis ((2-chloroquinolin-3-yl) methylene) benzene-1, 4-diamine with AKT1 was performed by using of GoldScore fitness function. The algorithm exhaustively searches the entire rotational and translational space of the ligand with respect to the receptors. The various solutions valuated by a score, which is equivalent to the absolute value of the total energy of the ligand in the protein environment. For each compound the best docking solutions of GOLD score was considered. GoldScore carry out a force field based scoring function and is made up of four components: 1) Ligand internal van der Waals energy (internal vdw); 2) Ligand intermolecular hydrogen bond energy (internal-H-bond); 3) Protein- ligand hydrogen bond energy (external H-bond); 4) Protein-ligand van der Waals energy (external vdw). When the total fitness score is computed the external vdw score is multiplied by a factor of 1.375. This is an empirical correction to persuade protein-ligand hydrophobic interaction. The fitness function has been optimized for the divination of ligand binding positions. GoldScore = S _(hb_ext)_ + S _(vdw_ext)_ + S _(hb_int)_ + S _(vdw_int)_, where S _(hb_ext)_ is the protein- ligand hydrogen bond score, S _(vdw___ext)_ is the protein-ligand Van der Waals score, S _(hb___int)_ is the score from intermolecular hydrogen bond in the ligand and S _(vdw___int)_ is the score from intermolecular strain in the ligand. Redocking of 1-(1-(4-(7-phenyl-1H-imidazo (4, 5-g) quinoxalin- 6-yl) benzyl) piperidin-4-yl)-1H-benzo[d]imidazol- 2(3H)-one (co crystal) ∆G = -44.43 and Ki = 1.64E-8, bonding model of the mentioned molecule was similar to which was reported in the crystal structure of 3O96. It was noted that GOLD scores of 3b and 3d are 113.76 and 107.58, respectively, which are greater than the other scores as shown in Table 2.

## Conclusion

 In summary we have described an efficient and convenient synthesis of N^1^, N^4^-bis ((2-chloroquinolin-3-yl) methylene) benzene-1, 4-diamine involving condensation reaction of 2- chloroquinoline-3-carbaldehydes and p-phenylenediamine in acidic condition. Docking was carried out using GOLD 5.2. It was found that both hydrogen bonding and hydrophobic interaction play important roles in the structure and function of biological molecules, peculiarly for inhibition in a complex. It was noted that GOLD scores of 3b and 3d are greater than the other scores.
